# Manual therapy versus therapeutic exercise in non-specific chronic neck pain: study protocol for a randomized controlled trial

**DOI:** 10.1186/s13063-019-3598-7

**Published:** 2019-08-09

**Authors:** Carlos Bernal-Utrera, Juan José González-Gerez, Manuel Saavedra-Hernandez, Miguel Ángel Lérida-Ortega, Cleofás Rodríguez-Blanco

**Affiliations:** 1 Doctoral Program in Health Sciences, University of Seville, Seville, Spain; 2 Fisiosur I+D Research Institute, Garrucha, Almería Spain; 3 Nursing, Physiotherapy and Medicine Department, Faculty of Health Sciences, University of Almería, Almería, Spain; 4 Health Sciences Department, Physiotherpy Area, Faculty of Health Sciences, University of Jaen, Jaen, Spain; 5 Physiotherapy Department, Faculty of Nursing, Physiotherapy and Podiatry, University of Seville, Seville, Spain

**Keywords:** Neck pain, Chronic pain, Exercise therapy, Musculoskeletal manipulations, Postural stability, Physical therapy specialty

## Abstract

**Background:**

The underlying mechanisms of non-specific chronic neck pain relapses are not clear, but they could be associated with a deficit and alteration of neck muscles propioception that play a decisive role in cervical joint position, motor control of the head, and postural stability. Numerous treatments for non-specific chronic neck pain have been described in the scientific literature. However, few studies analyze its influence on postural stability, since these alterations are not fully described, and various theories emerge about the reasons that cause it. Our primary aim is to analyze the differences in postural stability, pain, cervical disability, and the relation between them produced by a treatment based on manual therapy and another based on therapeutic exercise.

**Methods:**

The short-term and mid-term changes produced by different therapies on subjects with non-specific chronic neck pain will be studied. The sample will be randomly divided into three groups: manual therapy, therapeutic exercise, and placebo. As dependent variables of the study, we will take (1) Overall Balance Index, measured through a dynamic stabilometric platform; (2) pain, based on the visual analog scale and the Pressure Pain Threshold; (3) cervical disability, through the neck disability index. The findings will be analyzed statistically considering a 5% significance level (*p* ≤ 0.05).

**Discussion:**

Our study aims to provide knowledge about postural stability and its relationship with pain in subjects with non-specific chronic neck pain. Analyzing the results produced by different types of therapy will allow us to draw conclusions about the mechanisms, structural or central, that may elicit these alterations.

**Trial registration:**

Brazilian Clinical Trials Registry, RBR-2vj7sw. Registered on 28 November 2018.

**Electronic supplementary material:**

The online version of this article (10.1186/s13063-019-3598-7) contains supplementary material, which is available to authorized users.

## Background

Non-specific neck pain is pain that does not show pathognomonic signs and symptoms [1]. When the duration of symptoms is greater than 12 weeks of evolution, it acquires the value of chronicity and is denominated non-specific chronic neck pain (NCNP) [2]. It is a common disorder, which generates a great impact and socio-economic cost [3].

The underlying mechanisms of NCNP relapses are not clear, but the pain could be associated with a deficit and alteration of the proprioception of the neck muscles that play a decisive role in the cervical joint position, motor control of the head, muscles, and eyes, and postural stability (PS) [4–6].

Patients with NCNP usually have alterations in cervical proprioception and PS. They may also develop symptoms such as dizziness or vertigo [7, 8]. A recently published study shows that patients with NCNP suffer greater sensations of stunning and lack of proprioception than patients with benign paroxysmal vertigo [9].

Numerous studies downplay the efficacy of manual therapy and therapeutic exercise for pain reduction, cervical disability, and associated symptoms, such as dizziness [10–12]. However, there is less evidence of how these treatments, common in clinical practice, influence PS [13].

PS is highly influenced by the upper cervical spine and the suboccipital muscles, which are composed of up to 200 neuromuscular spindles per gram of muscle [14, 15]. This upper cervical segment is connected to the central nervous system (CNS), visual and vestibular apparatus, and sympathetic nervous system [16–19] in addition to cervical afferents through the cervico-ocular reflex (COR), the cervico-collic reflex (CCR), and the tonic neck reflex (TNR). The CCR activates the cervical musculature in response to stretching, maintaining good head position [20]; the COR acts through the vestibular reflex and the optokinetic reflex [21]. Finally, the TNR added to the vestibulospinal reflex achieves the maintenance of PS [22].

The alteration of this proprioceptive complex is not completely defined. Various theories have tried to explain how this system can be altered. Some studies indicate that there is a proprioceptive alteration due to sustained exposure to pain that affects PS through the CNS; these changes may be due to changes in the cortical representation and modulation of the cervical afferent contribution [23, 24]. In addition, some authors have begun to point out other psychobehavioral causes that could have a great influence on PS, such as anxiety, depression, or fear of movement [5, 6]. We must bear in mind that these variables are present in numerous patients with NCNP [25, 26].

However, other researchers relate the loss of PS to the dysfunction of the upper cervical spine and its musculature, changes in the cervical mechanoreceptors, and the state of weakness of the musculature [27–29], but these are not necessarily associated with traumatic events, since these types of alterations have been identified among subjects with NCNP without exposure to trauma [29].

The area of dizziness of cervicogenic cause is quite unknown; there are several theories about its cause, and there is no consensus on the diagnostic criteria [30]. More research is needed about relationships between neck pain, PS, and cervicogenic dizziness.

### Primary objective

The aim of our study is to compare two scientifically approved therapies for NCNP—one treatment with a greater influence on the structural component, and the other one with a greater component on the central process—to observe differences in the PS of the subjects with NCNP.

### Secondary objective

A secondary objective is to analyze the evolution of cervical pain and disability according to the treatment applied and the relationship with changes produced on PS.

### Hypothesis

Experimental treatments have a greater beneficial effect on PS and pain of subjects with NCNP than sham treatment. The improvement in PS is linked to an improvement in the subject’s pain.

### Trial design

This study is a randomized, controlled, parallel, double-blind, three-arm clinical trial of treatment.

## Methods/design

### Sample selection

Individuals with NCNP will be recruited through a text message broadcast on social networks in the city of Seville (Spain) and will be selected based on the eligibility criteria listed below. The study will take place in the facilities of the physiotherapy department of the University of Seville.

### Inclusion criteria

The inclusion criteria are:

- Age 18–50 years
- Current neck pain
- Neck pain continued for at least the last 12 weeks [2].

### Exclusion criteria

The exclusion criteria are:

- Irradiated neck pain
- Neck pain associated with vertigo
- Osteoporosis
- Psychological disorders
- Vertebral fractures
- Tumors
- Metabolic diseases
- Previous neck surgery
- Red flags (Night pain, severe muscle spasm, loss of involuntary weight, symptom mismatch)
- Physiotherapeutic treatment continued in the last 3 months

### Interventions

The participants can only receive the assigned treatment; they cannot combine the treatment with drugs or other physiotherapeutic treatment. Any interference in the treatment will be grounds for exclusion.

#### Group 1: manual therapy

The “manual therapy” protocol will consist of three techniques based on scientific evidence for the treatment of neck pain [31–33]. These techniques represent a very close approximation to the treatment that is performed in the daily clinic, outside the research protocols.

This protocol will be applied in the three treatment sessions, one per week.

1. High thoracic manipulation on T4 [31]
2. Cervical articular mobilization (2 Hz, 2 min × 3 series) [32]
3. Suboccipital muscle inhibition (3 min) [34].

#### Group 2: therapeutic exercise

The “therapeutic exercise” protocol will be taught to patients in the first session and should be done once a day during the 3 weeks of treatment. It will be reinforced by the physiotherapist in each of the three individual sessions.

##### Week 1. Exercises 1 and 2:

1. Cranio-cervical flexion (CCF) in supine position with towel in the posterior area of the neck (3 sets, 10 repetitions, 10 s of contraction each repetition with 10 s of rest)
2. CCF sitting (3 sets, 10 repetitions, 10 s of contraction each repetition with 10 s of rest)

##### Week 2. Exercises 1, 2, 3, and 4:

3. Co-contraction of deep and superficial neck flexors in supine decubitus (10 repetitions, 10 s of contraction with 10 s of rest)
4. Co-contraction flexors, rotators, and inclines. Patient will perfom cranial nerve flexion, while physiotherapist asks him to tilt, rotate, and look toward the same side while he opposes a resistance with his hand (10 repetitions, 10 s of contraction with 10 s of rest)

##### Week 3. Exercises 1, 2, 3, 4, 5, and 6:

5. Eccentric for extensors. With the patient seated, should perform cervical extension, then they must realize a CCF and then finish doing a cervical flexion (10 repetitions)
6. Eccentric for flexors. The patient will be in quadrupedal and neutral neck position. He should perform neck flexion, and then must realize a CCF and, maintaining that posture, extend the neck and then finally lose the CCF (10 repetitions).

#### Group 3: sham treatment

For the “control” protocol, the patient will be placed in the supine position, while the physiotherapist will lay his hands without therapeutic intention on the patient’s neck for 3 min, the physiotherapist will simulate the technique of suboccipital inhibition [34]. Later, with the laser pointer off, the patient will be contacted without exerting pressure for 10 s. Patients assigned to the control group will receive treatment 1 or 2 after completing the study.

### Outcome measures

#### Neck Disability Index (NDI)

The NDI is a self-assessment instrument of the specific functional status of subjects with neck pain with 10 elements, including pain, personal care, weight gain, reading, headache, concentration, work, driving, sleeping, and leisure. Each section is rated on a scale of 0 to 5, where 0 means “painless” and 5 means “the worst pain imaginable.” The points obtained are added to a total score. The questionnaire is interpreted as a percentage. The disability categories for NDI are 0–8%, without disability; 10–28%, mild; 30–48%, moderate; 50–64%, serious; and 70–100%, complete [35, 36].

#### Visual analog scale (VAS) for pain

The subjects participating in the study will indicate the intensity of their pain by means of a VAS of 100 mm. They must signal on a horizontal line of 100 mm where they would place their pain, where 0 mm indicated “no pain” and 100 mm would be “the worst pain imaginable” [37].

#### Pressure Pain Threshold (PPT)

The PPT is recorded in newtons/square centimeter using a digital algometer (Force Ten™ -Model FDX; Wagner, Greenwich, CT, USA) with a round tip surface area of 1 cm^2^. The measurement is taken on the spinous process of vertebra C2, the evaluator gradually increasing the pressure until the patient indicates through a “Yes” when the pain or discomfort appears. Three measurements are taken, obtaining an average value of these three measurements for the statistical analysis [38, 39].

#### Overall Balance Index (OBI)

We obtain the OBI measurement through a dynamic stabilometric platform (Balance System™ SD; Biodex, Shirley, NY, USA). The General Stability Test is applied at level of difficulty 4, with 1 being the most and 8 the least difficult level. The platform is free in the anterior-posterior and medial-lateral axes, and it allows one to obtain the OBI through deviations with respect to a zero point established before the test, with the platform stable. Two 20-s tests are performed, with 1 min between each test, with the score of the second test chosen for the statistical analysis. The index is calculated through the anterior-posterior and medial-lateral relationship + standard deviation [40, 41].

These variables will be measured in the pre-evaluation, first evaluation (week 2), second evaluation (week 4, short-term), and third evaluation (week 12, medium term). These evaluations will be carried out by an evaluator trained in these procedures, and the data will be stored in an Excel document.

### Participants’ timeline

**Fig. 1:**
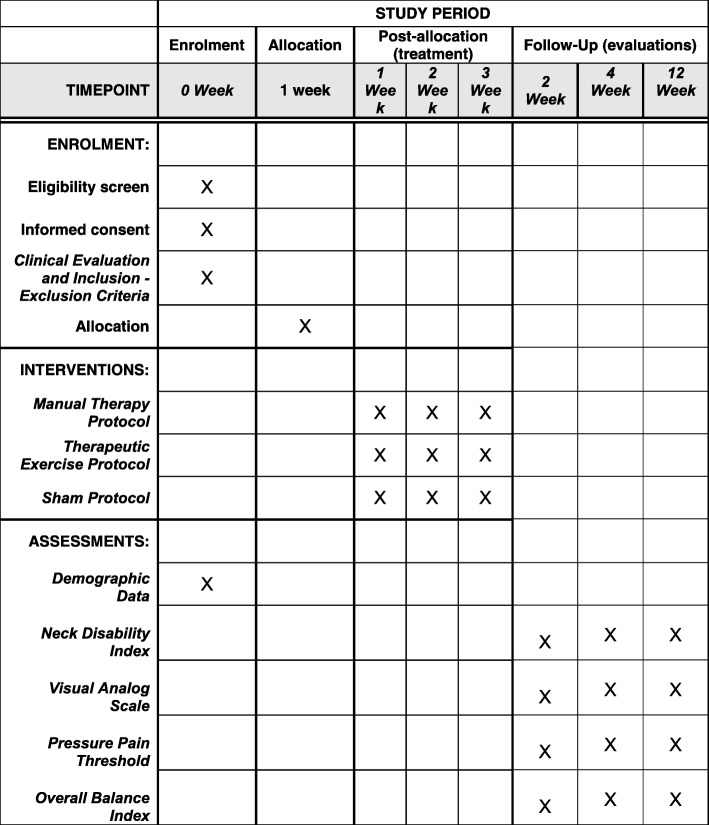
SPIRIT schedule for patient participation

A brief Standard Protocol Items: Recommendations for Interventional Trials (SPIRIT) schedule is provided in Fig. 1, and a populated SPIRIT checklist is provided in Additional file 1.

### Sample size calculation

The sample size was calculated using the Granmo calculator v.7.12. Based on the analysis of the variance of means, and estimating an alpha risk of 5% (0.05), a beta risk of 10% (0.10), a unilateral contrast, a typical deviation of 10% (0.10), a minimum difference to detect of 9.8% (0.098) which is based as the minimum clinically important differences in OBI [42], and a rate of follow-up losses of 15%, 10 subjects are required in each group, assuming that there are three groups. Finally, we will include 66 patients who will be divided into three groups, each group with at least 20 subjects, so as to overcome this value to assume the possible loss of follow-up.

### Randomization

Subjects will be divided into three groups by means of balanced randomization performed with free software (https://www.randomizer.org/). The randomization sequence will only be performed by the principal investigator and auditor.

### Blinding

The evaluator and participants in the study will be blinded during the entire process.

### Statistical analysis

The statistical analysis will be carried out using IBM-SPSS Statistics 24 software. The normality test applied to all the variables will be the Kolmogorov-Smirnov test. For the contrast of intragroup hypotheses, Student’s *t* test for paired variables will be applied in the case of parametric distributions and Kruskal-Wallis *H* for non-parametric distributions. For the intergroup hypothesis contrast, one-factor analysis of variance (ANOVA) will be used in the case of parametric distributions and Kruskal-Wallis *H* for non-parametric distributions. Post hoc analysis will be obtained through Bonferroni’s contrast for parametric distributions and Mann-Whitney’s *U* for non-parametric ones. Associations between pain (clinical improvement) and PS will be analyzed through Pearson’s *R* or Spearman’s rho. The confidence level used will be 95% (0.05), and the power of the study will be 90% (0.1).

## Discussion

This article presents a detailed description of a randomized controlled trial designed to analyze the results in terms of pain, disability, and postural stability of two types of treatments for non-specific chronic neck pain.

We intend to investigate a little-studied field such as postural stability in these subjects and to try to understand the mechanisms that may produce these alterations. We propose two types of treatments: one using manual therapy based on the structural influences of the neck, and another based on the therapeutic exercise that exerts its effect through more neurophysiological mechanisms. By observing the effects of these two therapies, we will try to analyze and gain a better understanding of the mechanisms that cause postural instability in patients with this type of pain. Our results intend to present whether the provocative mechanisms have a more structural component, or instead are caused by alterations produced at the level of the central nervous system by its sustained exposure to pain. In addition, we intend to establish relationships between clinical improvement in relation to pain with improvement in postural stability of the subjects and to analyze the differences depending on the treatment applied.

We have designed a randomized, controlled, double-blinded clinical trial, with the aim that our study can contribute to increase scientific knowledge on this matter and initiate new lines of future research.

### Trial status

This is the first and definitive protocol versión. Participants will be recruited between January and March 2019. Study completion is expected to be July 2019.

## Additional file

Additional file 1: SPIRIT Checklist. (DOC 122 kb)

## Data Availability

Not applicable.

## Abbreviations

CCF: Cranio-cervical flexion
NCNP: Non-Specific chronic neck pain
NDI: Neck Disability Index
OBI: Overall Balance Index
PPT: Pressure Pain Threshold
PS: Postural stability
VAS: Visual analog scale
